# Carbometallation chemistry

**DOI:** 10.3762/bjoc.9.27

**Published:** 2013-02-04

**Authors:** Ilan Marek

**Affiliations:** 1 Schulich Faculty of Chemistry, Technion-Israel Institute of Technology, Haifa 32000, Israel

**Keywords:** carbometallation

Following the pioneering Ziegler addition of nucleophiles to nonactivated unsaturated carbon–carbon bonds, the controlled carbometallation reaction has emerged. Since then, reactions that result in the addition of a carbon–metal bond of an organometallic across a carbon–carbon unsaturated system, leading to a new organometallic in which the newly formed carbon–metal bond can be used for further synthetic transformations, are called carbometallation reactions. In the past few decades, the intra- as well as intermolecular additions of various organometallic species to a large variety of alkynes, alkenes and allenes have been successfully reported. Although the carbometallation reaction on alkynes is generally a well-controlled and predictable transformation, leading to large variety of substituted stereocontrolled alkenyl metals, the addition of organometallic species to acyclic nonactivated alkenes still represents a formidable and yet unresolved synthetic challenge. Particularly stimulating would be the regio-, stereo- and enantioselective addition of organometallic species on α,β-disubstituted double bonds, leading to a configurationally stable sp^3^ organometallic that would subsequently react with electrophiles. This landmark transformation would formally result in the 1,2-bisalkylation of nonactivated alkenes! In this Thematic Series, you will find excellent contributions tackling various problems of carbometallation reactions, indicating a lively and rapidly moving field, and I have no doubt that more elegant transformations of C–C unsaturated bonds will continue to appear, including the enantioselective carbometallation reaction of substituted nonactivated alkenes. I would like to warmly thank all the contributors of this Thematic Series that have beautifully highlighted the state of the art of the field.

I have tremendously enjoyed reading this Thematic Series and I am convinced that you will all share in this pleasure!

Ilan Marek

Haifa, January 2013

